# When practice does not make a perfect - paradoxical learning curve in schizophrenia and bipolar disorder revealed by different serial reaction time task variants

**DOI:** 10.3389/fpsyt.2023.1238473

**Published:** 2023-09-12

**Authors:** Adrian Andrzej Chrobak, Katarzyna Siuda-Krzywicka, Zbigniew Soltys, Sylwia Bielak, Dominik Nowaczek, Aleksandra Żyrkowska, Magdalena Fafrowicz, Tadeusz Marek, Ewa Pęcherzewska, Jan Kużdżał, Anna Starowicz-Filip, Aleksandra Gorostowicz, Dominika Dudek, Marcin Siwek

**Affiliations:** ^1^Department of Adult Psychiatry, Jagiellonian University Medical College, Kraków, Poland; ^2^Faculté de Médecine, Sorbonne Université, Paris, France; ^3^Laboratory of Experimental Neuropathology, Institute of Zoology and Biomedical Research, Jagiellonian University, Kraków, Poland; ^4^Department of Adult, Child and Adolescent Psychiatry, University Hospital in Cracow, Kraków, Poland; ^5^J. Dietl Specialist Hospital, Kraków, Poland; ^6^Department of Cognitive Neuroscience and Neuroergonomics, Institute of Applied Psychology, Jagiellonian University, Kraków, Poland; ^7^Doctoral School in the Social Sciences, Jagiellonian University, Kraków, Poland; ^8^Faculty of Psychology, SWPS University of Social Sciences and Humanities, Katowice, Poland; ^9^Malopolska Centre of Biotechnology, Kraków, Poland; ^10^Medical Psychology Department, Jagiellonian University Medical College, Kraków, Poland; ^11^Department of Affective Disorders, Jagiellonian University Medical College, Kraków, Poland

**Keywords:** motor functions, movement disorders, cognition, cerebellum, procedural learning, affective disorders, sequence learning

## Abstract

**Introduction:**

Our previous studies identified a paradoxical implicit motor learning curve in schizophrenia (SZ) and bipolar disorder (BD) patients. This study aimed to verify whether those previously observed deficits may be captured by a new version of the ambidextrous serial reaction time task (SRTT), prepared for use in the MRI.

**Methods:**

This study involved 186 participants. A total of 97 participants (33 BD, 33 SZ, and 31 healthy controls, HCs) completed the original, unlimited time response variant of SRTT. A total of 90 individuals (30 BD, 30 SZ, and 30 HCs) underwent a newer, limited response time version of this procedure.

**Results:**

There was no significant difference in terms of implicit motor learning indices between both limited and unlimited response time SRTT. Compared to HCs, SZ, and BD patients presented decreased indices of implicit motor learning. Both clinical groups showed a paradoxical learning pattern that differed significantly from the HCs. Moreover, in the SZ group, the pattern depended on the hand performing SRTT.

**Discussion:**

The limited response time SRTT variant allowed us to replicate the findings of disrupted implicit motor learning in SZ and BD. The use of this paradigm in further neuroimaging studies may help to determine the neuronal underpinnings of this cognitive dysfunction in the abovementioned clinical groups.

## 1. Introduction

Throughout our lives, we acquire and retain motor skills that are crucial for the structurization of our behavior. Typing, riding a bike, or playing a musical instrument can be perfected through the repetition of a serial pattern of movements. Those skills can be acquired explicitly when the learner is aware of a motor sequence that makes up the task or implicitly when there is no conscious recollection of this pattern (1, 2). In neuropsychological research, the effectiveness of implicit learning can be assessed with the use of the serial reaction time task (SRTT) (3). During this procedure, participants are asked to respond to a stimulus presented on the screen by pressing a button corresponding to a given stimulus (e.g., the number 1 button in response to the same digit displayed on the screen). Unbeknownst to the subjects, the numbers are presented in a repetitive pattern. During the procedure, the participants implicitly learn the sequence, and their learning progress is reflected in shortening their reaction times (RT) with successive blocks of sequences. Then, after numerous repetitions of the learned sequence, the participants are presented with a block of randomly ordered numbers. This shift causes an increase (rebound) of the RT. The gradual decrease of the participants' RT to successive repetitions of sequences and the RT rebound associated with random stimulus presentation are indicators of implicit motor sequence learning.

It has been shown that this type of learning may be impaired in patients with mental disorders. Implicit motor learning deficits in schizophrenia (SZ) have been described in multiple studies and evaluated in the meta-analysis of Siegert et al. (4). Recently, we have shown that those impairments are also present in bipolar disorder (BD) patients (5). In this study, we have identified that SZ and BD patients present peculiar implicit motor learning impairments (5, 6). Both clinical groups showed a paradoxical learning curve. While healthy individuals presented the neurotypical learning process in which RT gradually decreased with the repetition of the sequence and increased with the presentation of random stimuli, the patients revealed a somewhat opposite pattern. Their tapping speed became slower in the last block with the sequence and then increased when the random stimuli appeared (reversed rebound). Thus, the execution of the SRTT exhibited the features of an inverted learning curve. Moreover, in the group of SZ patients, the course of implicit motor learning depended on the hand performing the task. SZ individuals presented more severe deficits during SRTT when performing with their left hand. We have hypothesized that our findings could be associated with the structural and functional dysfunctions of the cerebellum and its networks, as observed in SZ and BD (5–7).

A growing body of evidence shows that SZ and BD patients may share common neurodevelopmental and genetic abnormalities, that may result in an overlap of neuropsychological and motor function deficits and their neurobiological substrates (8). There is scarce research on neuronal underpinnings of implicit motor learning deficits in the abovementioned clinical groups. Only three fMRI studies evaluated SRTT performance in SZ patients (9–11). All of them failed to replicate previous findings of implicit motor learning impairments in this group. These discordances were linked with the differences between SRTT procedures used in the behavioral and MRI studies discussed thoroughly in (12). Only one study performed a neuroimaging evaluation of implicit learning in BD patients (13). Unfortunately, the repetitive sequence used in the SRTT in that study was relatively simple, and most of the subjects were able to notice when it changed. Thus, participants developed explicit knowledge during the task of what significantly hinders the evaluation of implicit motor learning (5, 13). Noteworthy, none of the abovementioned studies evaluated whether SRTT performance is associated with the hand performing the task.

The current research aimed to adapt the ambidextrous SRTT procedure that we have used in our previous studies to be used in fMRI. In our original SRTT procedure, the participants had unlimited time to respond, which is impossible to maintain the settings of a functional MRI experiment. To adapt our procedure to the temporal requirements of fMRI methods, we restricted the subjects' response time to standardize the duration of the task for each participant. The goal of our current study was to replicate our previous findings of implicit motor learning deficits in SZ and BD patients in the context of response time constraints.

## 2. Methods

### 2.1. Participants

This study involved 186 participants. A total of 90 subjects performed limited-time response SRTT. They were 30 BD patients (12 Bipolar I and 18 Bipolar II patients), 30 SZ patients, and 30 healthy controls (HCs). The group of 97 participants who completed unlimited time response SRTT was described in our previous study (6). It consisted of 33 BD patients (15 Bipolar I and 18 Bipolar II patients), 33 SZ patients, and 31 HCs. Participants performing limited time response SRTT were recruited according to the methodology of (6) the study to directly compare their implicit motor learning performance with the unlimited time response group. The diagnosis and clinical assessment of the participants were done by an experienced psychiatrist according to ICD-10 and DSM-5 criteria. The inclusion criteria for SZ and BD groups were the state of symptomatic remission [three or fewer on all PANSS items, and in the case of BD patients—the state of euthymia defined as <11 points in the Montgomery–Åsberg Depression Rating Scale (14) and <5 points in the Young Mania Rating Scale (15)] and treatment with the antipsychotic drugs from the group of dibenzoxazepine (clozapine, olanzapine, or quetiapine). The selection of the atypical antipsychotics from this group provided a relative pharmacological homogeneity across BD and SZ patients and minimized the treatment effect on patients' motor performance. Individuals treated with lithium were excluded as the treatment with this drug may have an impact on cerebellar functions (16). Additional treatment with the use of valproic acid and lamotrigine was accepted. In order to increase the clinical homogeneity of studied groups, the following exclusion criteria were implemented for the patients: (a) history of drug or alcohol abuse according to the substance use disorder of DSM-5; (b) acute, severe, or chronic somatic and neurological diseases; (c) severe personality disorders; and (d) treatment other than listed in the inclusion criteria. HCs were recruited *via* the author's social network. They were interviewed by an experienced psychiatrist. All HCs reported negative history of mental and neurological disorders and did not meet any exclusion criteria for patients. All participants were right handed, as measured by the Neurological Evaluation Scale (17). All three groups were matched for gender and age. Patients were matched in terms of the duration of their treatment. The demographic characteristics of the studied groups are presented in Table 1. All participants signed and informed written consent prior to the assessment. The study was approved by the Jagiellonian University Bioethics Committee.

**Table 1:** Description of examined groups.

**SRTT type**	**Limited response time**			**Unlimited response time**			**Main effect for the SRTT variant**		**Main effect for the group**	
**Groups**	**SZ**	**BD**	**HC**	**SZ**	**BD**	**HC**	***F*****(1, 181) or** ***F*****(1, 122)**^†^	**Effect size**	***F*****(2, 181) or** ***F*****(1, 122)**^†^	**Effect size**
Age [mean (SD)]	40.57 (12.38)	38.3 (11.88)	39.73 (11.63)	36.39 (11.96)	42.45 (13.57)	38.03 (11.09)	0.104	< 0.001	0.426	0.005
Sex (men/women)^§^	12/18	18/12	16/14	16/17	23/10	16/15	–	–	–	–
Years of education [mean (SD)]	15.3 (2.67)	16.27 (2.68)	15.33 (2.12)	14.48 (2.58)	15.15 (2.55)	16.13 (1.8)	1.103	0.006	2.231	0.026
Duration of treatment	10.47 (6.75)	8.2 (6.98)		10.27 (7.89)	11.38 (7.4)		1.318	0.011	0.199	0.002
Equivalent of olanzapine daily dosage [mean mg/day, (SD)]				14.23 (5.24)	10.37 (6.32)					
BD type (I/II)^#^	–	13/17	–	–	15/18	–	–	–	–	–
Equivalent of olanzapine daily dosage [mg/day, (SD)]				14.23 (5.24)	10.37 (6.32)					

SRTT, serial reaction time task; SZ, schizophrenia; BD, bipolar disorder; HCs, healthy controls; SD, standard deviation. Effect size (generalized eta squared) lower than 0.01 was counted as negligible, 0.01–0.06 as small, 0.06–0.14 as medium, and higher than 0.14 as large.

${\;}^{\text{\#}}\chi_{\left(2,63\right)}^{2}$ = 0.0286, *p* = 0.87.

§ χ^2^ comparisons of gender distribution among following groups: limited response time groups $\chi_{\left(2,90\right)}^{2}$ =2.49, *p* = 0.28, unlimited response time group $\chi_{\left(2,97\right)}^{2}$ = 3.5, *p* = 0.173; unlimited and limited response time groups combined, $\chi_{\left(2,187\right)}^{2}$ = 5.39, *p* = 0.06.

† F(1, 181) and F(2, 181) refers to comparison of SZ, BD, and HC groups, while F(1, 122) applies to the analysis of BD and SZ groups.

### 2.2. Serial reaction time task procedures

**Figure 1:**
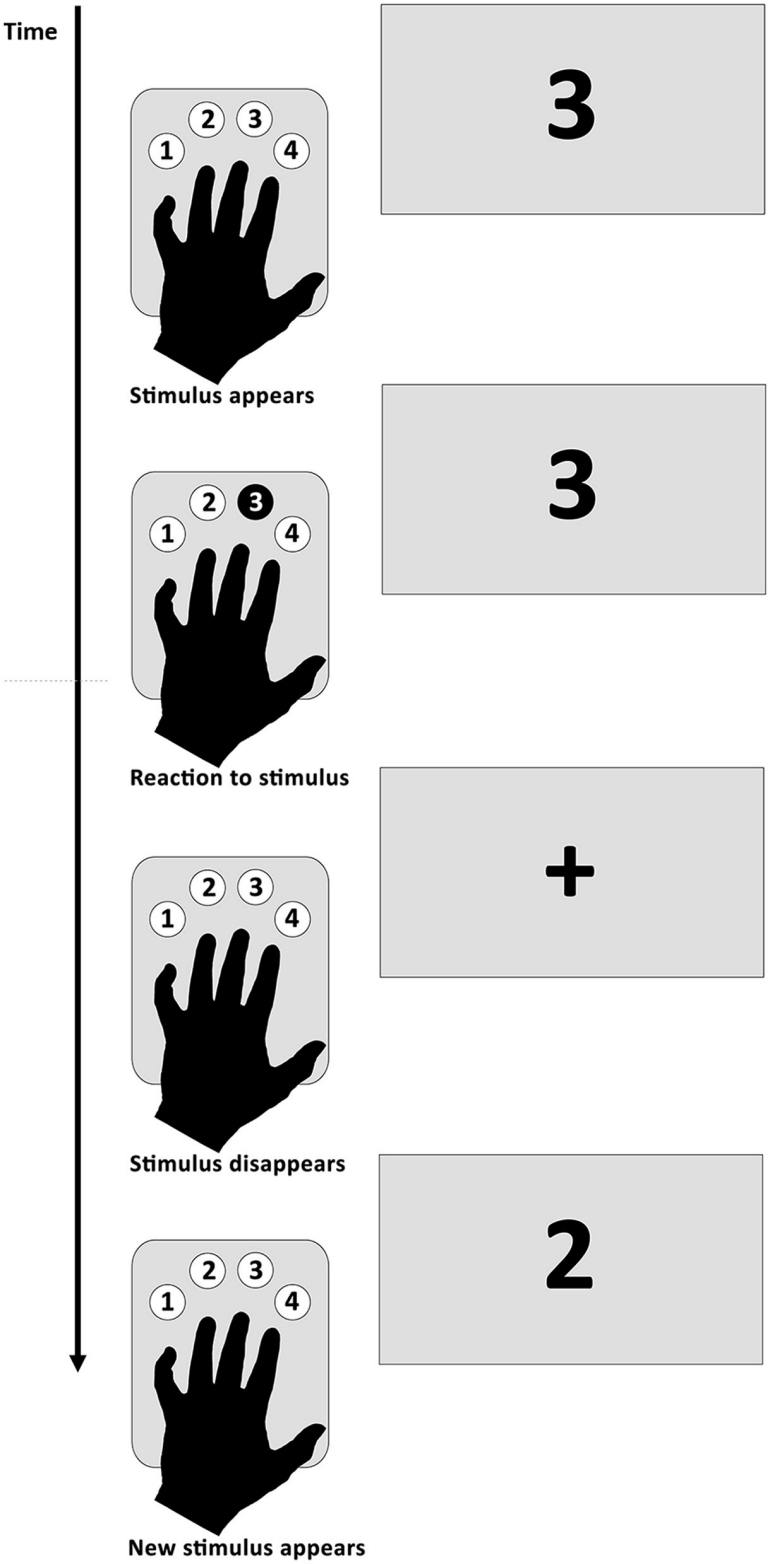
Serial reaction time task procedure.

Both limited and unlimited response time SRTT were adopted by Gómez-Beldarrain et al. (18). The latter procedure was used in our previous studies (5, 6). Participants performed the task with their left and right hands separately with the use of the response tab containing four buttons numbered from 1 to 4 (Figure 1). In the case of the right hand, subjects were instructed to put their index finger on the button number 1, middle finger on the button number 2, ring finger on the button number 3, and little finger on the button number 4. In the case of the left hand, the order was reversed. Participants were asked to respond as fast and as accurately as possible by pressing the button corresponding to the number displayed on the screen. The procedure consisted of five blocks. In blocks 2–4, the participants were presented with 10 series of 10-item identical sequences of numbers from 1 to 4. The numbers in the sequence were not predictable, and a given number was never followed by the same one. Two different sequences were used for the left and the right hand to avoid cross-hand learning. The sequences were counterbalanced across subjects so that half of them started with sequence number 1 and the rest with sequence number 2. In blocks 1 and 5, the order of the numbers from 1 to 4 was random. No breaks were made between the blocks during the procedure. After finishing the task, participants were asked if they could identify the presence of the sequence, and if so, they were asked to recall it (5–7).

#### 2.2.1. Unlimited response time SRTT variant

The methodology of this variant was adapted from (18) and was used in our previous studies (5–7). During this SRTT variant, participants sat in front of a computer screen. All of them performed the task first with their right and then with their left hand. Participants had unlimited time to respond to the number displayed on the screen with the suitable button on the response pad. When the response was correct, the number disappeared from the screen (response triggered offset) and the next one was presented after 500 ms. The duration of the procedure was dependent solely on the time of reaction of the subjects. The task took an average of 1 h 32 min in the SZ group, 1 h 17 min in the BD group, and 50 min in the HC group (5–7).

#### 2.2.2. Limited response time SRTT variant

During this SRTT variant, participants were lying on their back inside of the MRI scanner. Half of them performed the task first with their right hand and the rest with their left hand. When the number was displayed on the screen, participants had 1.5 s to respond with an adequate number, and the next one was presented after the pseudorandomized time (400, 500, or 600 ms). The duration of the whole task was 34 min.

### 2.3. Data analysis

A data analysis strategy was adopted from (18) and was used in our previous studies (5–7). RT was calculated as the time between the appearance of a number on the screen and the first button press. The median of RT (mRT) was calculated for each of the five blocks. In order to normalize participants' baseline performance, mRT from blocks 2–4 was divided by the mRT from block 1 and multiplied by 100% (mRT as a percent of the mRT of block number 1, mRT%). This conversion allowed us to compare differences in the learning dynamic despite the discrepancies in RT between subject groups. Two major indices were chosen to reflect implicit motor learning tasks. The first is the decrease of mRT% across the first and the last block containing the sequence (difference between mRT% of blocks no. 2 and 4). The second one, rebound, reflects the difference in mRT% between the last block consisting of the sequence and the final block comprising numbers in the random order (mRT% in block 5—mRT% in block 4). The rebound indicates that the mRT% decrease across the blocks is associated with the presence of the sequence (7).

Statistical analyses were made in R software (19). Demographic characteristics were compared with the use of one-way ANOVA and χ^2^ tests as appropriate. The Levene test was used to evaluate the homogeneity of variance. Three-way ANOVA, followed by effect size calculation (eta-squared), was used to evaluate the effect of the SRTT variant (limited or unlimited response time), group (SZ, BD, or HC) and hand (left or right) on the indices of implicit motor learning (the difference between mRT% of blocks 2 and 4 and the rebound) and mRT% of block 1–5. Two-way ANOVA was used to evaluate the effect of the group and the hand on the indices of implicit motor learning (the difference between mRT% of blocks no. 2 and 4 and the rebound) and mRT% of blocks 1–5 during the limited response time SRTT variant. The daily dosage of antipsychotics was converted to the equivalent of olanzapine according to Leucht et al. (20). Associations between demographic and clinical variables (age, years, education, equivalent of the daily dose of olanzapine, and the duration of treatment) and the indices of implicit motor learning were calculated by a series of simple linear regression analyses. ANOVA, effect size, and *post-hoc* comparison were performed using the rstatix package. For other analyses, functions from the stats package were used. For the visualization of the results, ggplot2 and ggpubr packages were used.

## 3. Results

**Figure 2:**
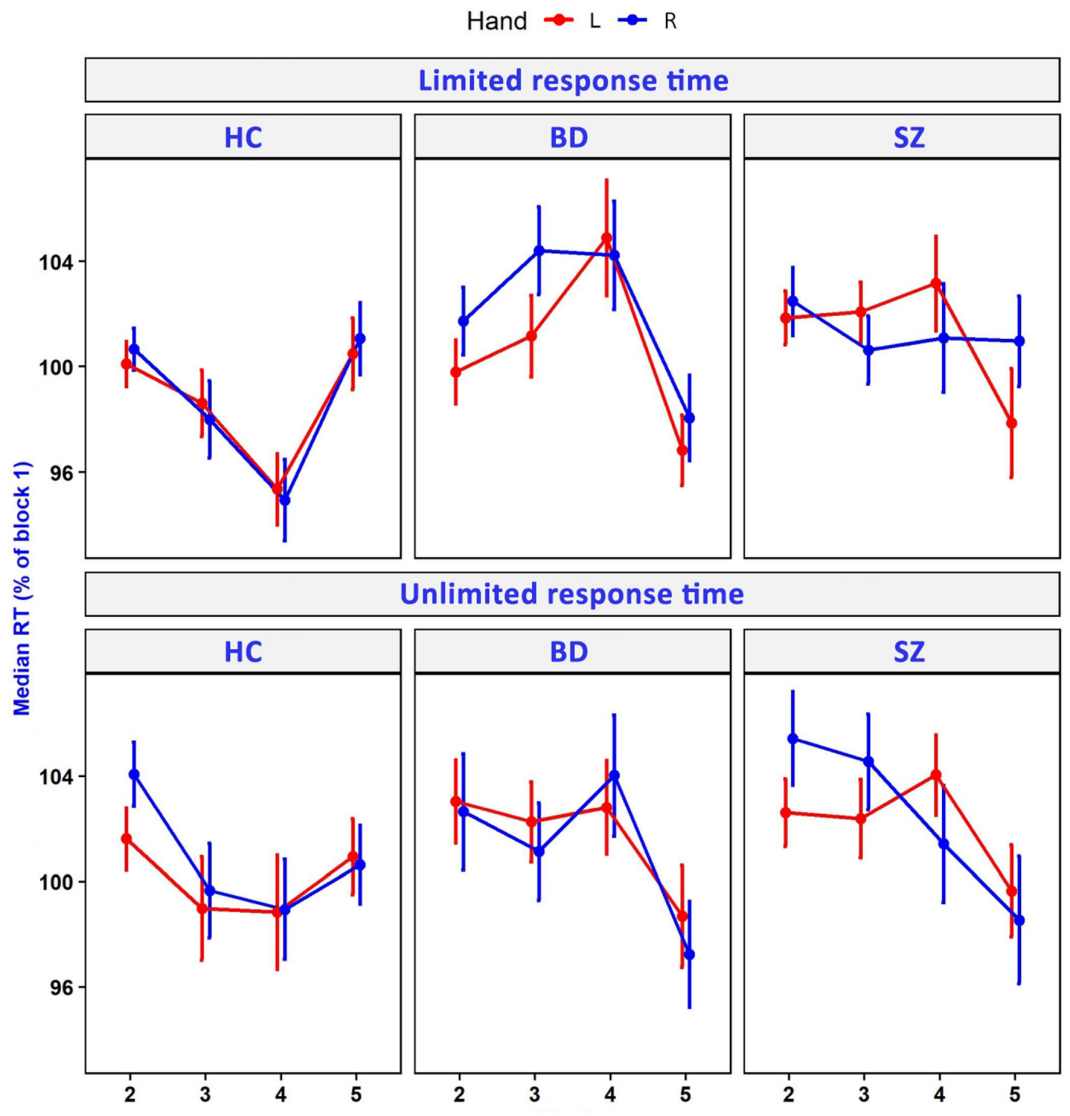
Median reaction time (RT) in consecutive blocks of unlimited and limited response time variants of SRTT. RT has been expressed as % of RT in block 1. HCs, healthy controls; BD, bipolar disorder; SZ, schizophrenia; L, left; R, right.

**Figure 3:**
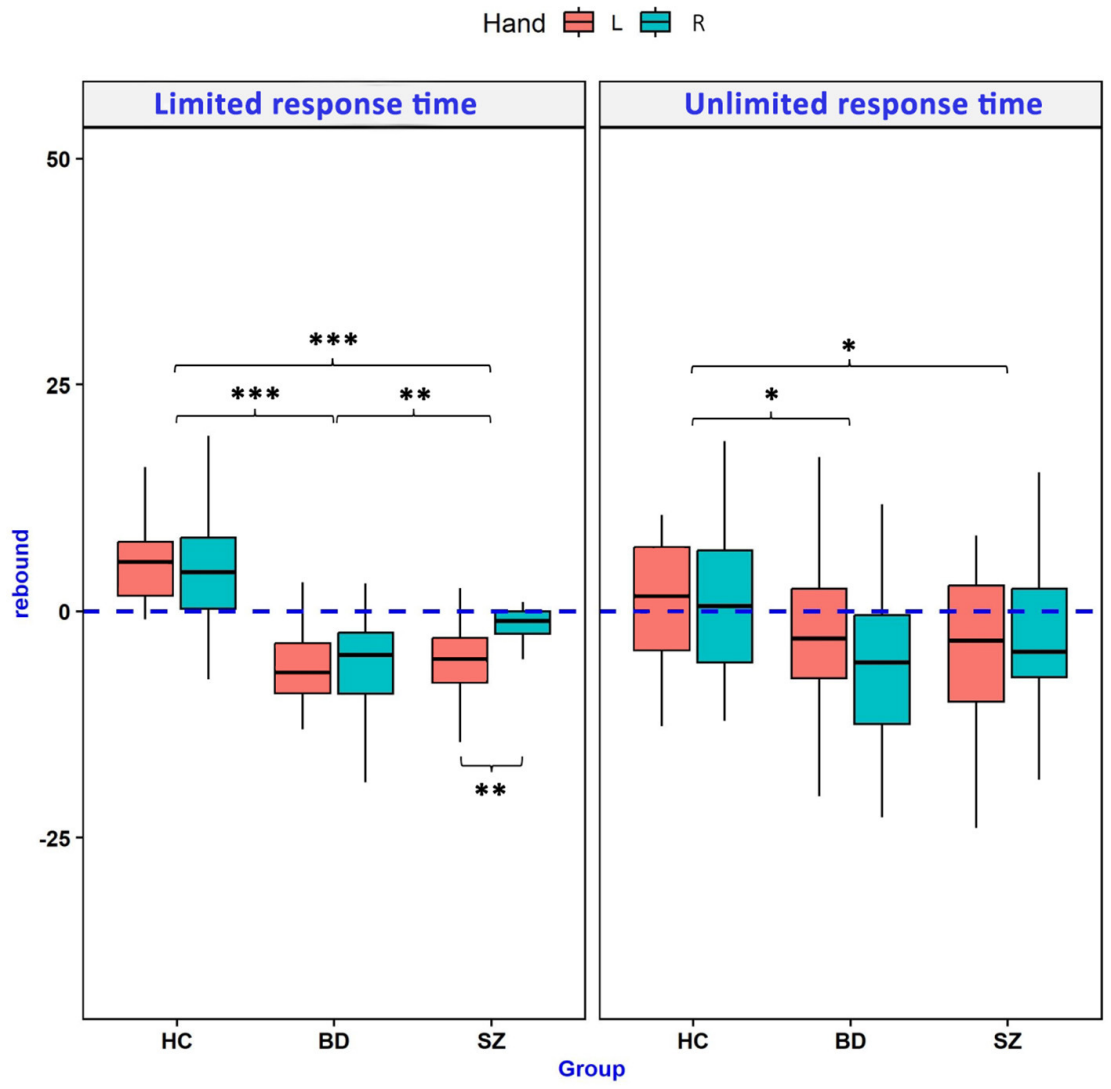
Comparison of the RT rebound between groups performing limited and unlimited response time variants of the serial reaction time task. Positive rebound values are the indicators of implicit motor learning in this paradigm. Asterisk denotes the statistical significance of two-way ANOVA *post-hoc* tests (Tukey or Games-Howell tests according to the homogeneity of variance). HCs, healthy controls; BD, bipolar disorder; SZ, schizophrenia; L, left; R, right. *p ≤ 0.05, **p ≤ 0.01, ***p ≤ 0.001.

There was no significant effect of the SRTT variant (limited vs. unlimited time to respond) on the indices of implicit motor learning. The SRTT variant was related only to the mRT% of block 2. Diagnosis (SZ, BD, and HC) was associated with the indices of implicit motor learning as well as mRT% of blocks 3 and 4. Additionally, the difference between mRT% of blocks 4 and 2 was dependent on the hand performing the task. Table 2 presents the details of this analysis. Comparison of mRT% across blocks 2–5 of two SRTT variants for SZ, BD, and HC groups is presented in Figure 2. Comparison of rebound values is illustrated in Figure 3.

**Table 2:** Results of the three-way ANOVA analysis evaluating the effects of serial reaction time task (SRTT) variants (limited vs. unlimited response time), studied group (schizophrenia, bipolar disorder, and healthy controls), and hand (left vs. right) on the indices of implicit motor learning and normalized medians of reaction times (mRT%) in blocks 2–5.

	**Main effect for the SRTT variant**		**Main effect for the diagnosis**		**Main effect for the hand**	
	***F*** **(1, 181)**	**Effect size**	***F*** **(2, 181)**	**Effect size**	***F*** **(1, 181)**	**Effect size**
**Indices of implicit motor learning**						
Difference between mRT2% and mRT4%	1.231	0.004	15.108^***^	0.09	6.941^**^	0.015
Rebound	1.457	0.004	37.996^***^	0.182	1.509	0.004
**Normalized medians of reaction times in blocks 2–5**						
mRT2%	8.940^*^	0.027	2.594	0.016	3.215	0.008
mRT3%	1.376	0.004	7.097^*^	0.04	0.340	< 0.001
mRT4%	1.448	0.005	12.208^***^	0.073	0.587	0.001
mRT5%	0.083	0.000276	2.414	0.016	0.112	< 0.001

Effect size (generalized eta squared) lower than 0.01 was counted as negligible, 0.01–0.06 as small, 0.06–0.14 as medium, and for >0.14 as large.

* *p* ≤ 0.05,

** *p* ≤ 0.01,

*** *p* ≤ 0.001.

The limited response time SRTT variant showed significant differences between the studied Groups. In comparison to HCs, SZ, and BD patients showed decreased measures of both implicit motor learning indices (rebound and the difference between mRT% in blocks 2 and 4) as well as increased mRT% in blocks 3 and 4. Moreover, BD patients presented more severe implicit motor learning deficits than the SZ group in terms of lower values of rebound and the difference between mRT% in blocks 2 and 4. In the SZ group, SRTT results differed depending on the hand performing the task. Patients revealed significantly lower rebound values when they were using the left hand. Detailed results of a two-way ANOVA analysis are presented in Table 3.

**Table 3:** Results of the two-way ANOVA analysis evaluating differences between the schizophrenia, bipolar disorder, and healthy controls groups in terms of indices of implicit motor learning and normalized medians of reaction times (mRT%) in blocks 2–5 measured by the limited response time serial reaction time task variant.

	**Main effect for the group**		**HC vs. BD**	**HC vs. SZ**	**BD vs. SZ**	**Main effect for the hand**		**BD**	**SZ**	**HC**	**Interaction**	
	***F*** **(2, 87)**	**Effect size**				***F*** **(1, 87)**	**Effect size**				***F*** **(2, 87)**	**Effect size**
**Implicit motor learning indices**												
Difference between mRT2% and mRT4%	20.684^***^	0.186	*p* < 0.001	*p* < 0.001	*p* = 0.008	3.072	0.018	–	–	–	0.220	0.003
Rebound	43.119^***^	0.332	*p* < 0.001	*p* < 0.001	*p* = 0.002	5.567^***^	0.031	–	*p* = 0.003	–	1.240	0.014
**Normalized medians of reaction times in blocks 2-5**												
mRT2%	1.257	0.017	–	–	–	1.633	0.008	–	–	–	0.303	0.003
mRT3%	5.122^**^	0.058	*p* = 0.005	*p* = 0.0471	–	0.126	< 0.001	–	–	–	1.666	0.018
mRT4%	12.478^***^	0.137	*p* < 0.001	*p* < 0.001	–	0.537	0.003	–	–	–	0.128	0.001
mRT5%	1.815	0.025	–	–	–	1.993	0.009	–	–	–	0.427	0.004

BD, bipolar disorder; SZ, schizophrenia; HCs, healthy controls. Effect size lower than 0.01 was counted as negligible, 0.01–0.06 as small, 0.06–0.14 as medium, and for >0.14 as large. *P*-values for the main effects were adjusted for multiple comparisons (Benjamini–Hochberg correction).

** *p* ≤ 0.01;

*** *p* ≤ 0.001.

Regression analyses showed no significant associations between age, years of education, duration of treatment, the daily dose of olanzapine equivalent, and implicit motor learning indices within groups evaluated with the use of limited response time SRTT. Detailed results of those analyses are presented in Table 4.

**Table 4:** Associations between demographic and clinical variables and indices of implicit motor learning measured by limited response time serial reaction time task variant.

	**Indices of implicit motor learning**	**Group**	**Hand**	**β**	***R^2^***
Age	Difference between mRT2% and mRT4%	HC	L	0.004	0
			R	0.147	0.044
		BD	L	−0.142	0.029
			R	0.145	0.052
		SZ	L	0.035	0.004
			R	0.045	0.004
	Rebound	HC	L	−0.005	0.0002
			R	0.083	0.011
		BD	L	−0.07	0.009
			R	0.077	0.015
		SZ	L	0.049	0.011
			R	−0.074	0.01
Years of education	Difference between mRT2% and mRT4%	HC	L	0.015	0
			R	0.855	0.049
		BD	L	0.495	0.018
			R	0.333	0.014
		SZ	L	0.549	0.046
			R	0.797	0.063
	Rebound	HC	L	−0.139	0.005
			R	0.837	0.039
		BD	L	0.647	0.04
			R	−0.361	0.016
		SZ	L	−0.079	0.001
			R	−0.012	0
Duration of treatment	Difference between mRT2% and mRT4%	BD	L	−0.002	0
			R	−0.052	0.002
		SZ	L	−0.068	0.004
			R	0.048	0.001
	Rebound	BD	L	−0.028	0.0005
			R	0.098	0.008
		SZ	L	0.066	0.006
			R	−0.237	0.03
Daily dose of olanzapine equivalent	Difference between mRT2% and mRT4%	BD	L	0.389	0.051
			R	−0.052	0.001
		SZ	L	−0.166	0.022
			R	0.03	0
	Rebound	BD	L	0.396	0.07
			R	−0.076	0.003
		SZ	L	−0.21	0.047
			R	0.362	0.058

BD, bipolar disorder; SZ, schizophrenia; HCs, healthy controls; mRT%, normalized medians of reaction times; R, right; L, left. All associations were not statistically significant.

A detailed description of the results obtained from the analyses of the unlimited response time SRTT variant has been described in our previous studies (5–7).

## 4. Discussion

To the best of our knowledge, this is the largest study of implicit motor learning abnormalities in BD and SZ. We have shown that BD and SZ patients present implicit learning deficits in both unlimited and limited response time variants of the ambidextrous SRTT. The use of the limited response time SRTT replicated our previous findings showing a paradoxical learning curve in both disorders as well as in unilateral deficits of this cognitive function in SZ.

This is the first study comparing different variants of SRTT in SZ and BD. We have shown no significant impact of the SRTT variant on the indices of implicit motor learning in the studied groups. Both methods enabled the observation of the typical learning curve in the HC group, expressed by positive values of rebound and the difference between mRT4% and mRT2% parameters. The only parameter influenced by the SRTT variant was mRT2%; however, despite this association, there was no difference between both methods in terms of implicit motor learning indices. Our results indicate that both types of SRTT can be used interchangeably to assess impairment of this cognitive function. The ambidextrous, limited time response SRTT variant described in our study may be useful in future research in which the duration of the task must be equal for all of the participants, notably in the fMRI studies.

In his critical review of the literature, Remillard (12) comprehensively analyzed studies evaluating implicit motor learning in SZ with the use of SRTT. The author found that every study that used response-triggered offset, where a response to the onset of the target immediately leads to its offset, found impaired sequence learning in the SZ patient group. On the contrary, every study that has used a time-triggered offset, where the offset of the target occurs after a fixed amount of time has elapsed since its onset, did not find the difference between SZ and HC groups. All fMRI studies evaluating implicit motor learning with the use of SRTT in this clinical group used the latter version of this task, indicating normal sequence learning in SZ (9–11). In our study, both limited and unlimited response time SRTT variants had response-triggered offset, which revealed significant deficits of this cognitive function in SZ and BD patients' groups. Thus, we recommend that future studies that aim to analyze implicit motor learning deficits in those clinical groups should use response-triggered offset variants of SRTT.

Analysis of the limited response time SRTT showed that both SZ and BD patients groups present implicit motor learning deficits compared to HCs. With the use of this paradigm, we have replicated the results of our previous studies, indicating the presence of the paradoxical learning curve in both clinical groups and the more pronounced motor learning deficits when the task was performed with the left hand in the SZ patient group. However, in our previous study, this unilateral deficit was reflected by the lack of the RT decrease between blocks 2 and 4, while in limited time response SRTT, this impairment concerned the rebound. Moreover, a limited response time SRTT variant showed more severe implicit motor learning in BD compared to the SZ patients' group. Our previous study showed that while BD patients show no signs of implicit sequence learning, SZ patients exhibit some features of motor learning when using the right hand, reflected by a gradual decrease in RT across all of the SRTT blocks. Both disorders share structural and functional impairments of the brain areas involved in SRTT (2), such as the prefrontal cortex (21, 22), primary motor cortex (23–25), supplementary motor area (26–28), cerebellum (29, 30), and striatum (11, 31, 32). Moreover, SZ and BD patients share motor function deficits associated with the abovementioned structures impairments, such as neurological and cerebellar soft signs (33–35), eyeblink conditioning (cerebellar learning paradigm) (36, 37), or oculomotor deficits (8, 38, 39). Further studies with the use of ambidextrous response triggered offset SRTT are needed to establish differences between SZ and BD in terms of implicit motor learning deficits and their neuronal underpinnings.

We are aware of several limitations of our study. While this is the largest implicit motor learning study in SZ and BD, the number of subjects in analyzed subgroups was relatively low. Patients were not drug-naïve, and we have not measured potential antipsychotic-induced movement disorders in these groups. However, as in our previous studies, we have observed no significant associations between antipsychotic drug dose and implicit motor learning indices. Moreover, we have found no effect of confounding factors such as duration of treatment, age, gender, and years of education on SRTT performance. An important limitation factor in the case of comparison of limited and unlimited response time SRTT is the fact that the tasks were performed by the different subject groups. However, this limitation is unavoidable because once the participants completed the task, they are assessed on whether they consciously recognized the presence of the sequence. Without this evaluation, it would not be possible to verify whether the motor learning process was implicit. Presumably, repeating the task by the participant that previously completed one of the SRTT variants would have an impact on subsequent measures with the use of this paradigm as the subject already learned the sequence and gained proficiency in handling response buttons. Moreover, SRTT variants compared in this study differed not only in terms of the response time restriction but also in the presence of pseudorandomization of the pauses between the presentations of the stimuli, counterbalancing the effect of hand with which the procedure was started and the participants' position (sitting vs. lying on the back). Nevertheless, our results showed a lack of differences in terms of implicit motor learning indices between evaluated SRTT variants. A comprehensive discussion of other potential effects of medication as well as cognitive and behavioral dysfunctions on SRTT performance has been presented in our previous study (6).

In conclusion, our study provides further evidence of implicit motor learning impairments in BD and SZ. Both disorders present a paradoxical learning curve reflected by the negative rebound values. Moreover, in the group of SZ, those impairments differ depending on the hand performing SRTT. There is no significant difference in terms of implicit motor learning indices between both limited and unlimited response time SRTT. Both methods capture correct learning curves in the HC group. The ambidextrous SRTT variant with response-triggered offset and limited response time described in our study is valid to evaluate implicit motor learning impairments in SZ and BD patients. The use of this paradigm in further neuroimaging studies may help to determine the neuronal underpinnings of this cognitive dysfunction in the abovementioned clinical groups.

## Data availability statement

The raw data supporting the conclusions of this article will be made available by the authors, without undue reservation.

## Ethics statement

The studies involving humans were approved by Jagiellonian University Bioethics Committee, Jagiellonian University Medical College, Kraków, Poland. The studies were conducted in accordance with the local legislation and institutional requirements. The participants provided their written informed consent to participate in this study.

## Author contributions

AC: conceptualization and writing—original draft preparation. AC, KS-K, MF, DD, and MS: methodology. ZS and AC: formal analysis, data curation, and visualization. AC, JK, AŻ, AG, EP, SB, and DN: investigation. AC, MF, and TM: resources. AC, JK, AŻ, AG, EP, ZS, DD, MS, MF, TM, DN, and SB: writing—review and editing. DD and MS: supervision. DD, MS, and AC: project administration. AC, MF, and TM: funding acquisition. All authors have read and agreed to the published version of the manuscript.

## References

[1] Willingham DB Goedert-Eschmann K. The relation between implicit and explicit learning: evidence for parallel development. Psychol Sci. (1999) 10:531–4. 10.1111/1467-9280.00201

[2] Tzvi E Münte TF Krämer UM. Delineating the cortico-striatal-cerebellar network in implicit motor sequence learning. Neuroimage. (2014) 94C:222–30. 10.1016/j.neuroimage.2014.03.004 24632466

[3] Chrobak AA Tereszko A Jeziorko S Siwek M Dudek D. Paradigms of procedural learning - a review of selected methods. Neuropsychiatr Neuropsychol. (2014) 9:62–70.

[4] Siegert RJ Weatherall M Bell EM. Is implicit sequence learning impaired in schizophrenia? A meta-analysis. Brain Cogn. (2008) 67:351–9. 10.1016/j.bandc.2008.02.005 18378373

[5] Chrobak AA Siuda-Krzywicka K Siwek GP Arciszewska A Siwek M Starowicz-Filip A. Implicit motor learning in bipolar disorder. J Affect Disord. (2015) 174:250–6. 10.1016/j.jad.2014.11.043 25527995

[6] Chrobak AA Siuda-Krzywicka K Siwek GP Tereszko A Janeczko W Starowicz-Filip A. Disrupted implicit motor sequence learning in schizophrenia and bipolar disorder revealed with ambidextrous Serial Reaction Time Task. Prog Neuropsychopharmacol Biol Psychiatry. (2017) 79(Pt B):169–75. 10.1016/j.pnpbp.2017.06.025 28648566

[7] Chrobak AA Siuda-Krzywicka K Sołtys Z Siwek GP Bohaterewicz B Sobczak AM. Relationship between neurological and cerebellar soft signs, and implicit motor learning in schizophrenia and bipolar disorder. Prog Neuropsychopharmacol Biol Psychiatry. (2021) 111:110137. 10.1016/j.pnpbp.2020.110137 33053417

[8] Ivleva EI Morris DW Moates AF Suppes T Thaker GK Tamminga CA. Genetics and intermediate phenotypes of the schizophrenia–bipolar disorder boundary. Neurosci Biobehav Rev. (2010) 34:897–921. 10.1016/j.neubiorev.2009.11.022 19954751 PMC12779254

[9] Zedkova L Woodward ND Harding I Tibbo PG Purdon SE. Procedural learning in schizophrenia investigated with functional magnetic resonance imaging. Schizophr Res. (2006) 88:198–207. 10.1016/j.schres.2006.06.039 16945506

[10] Purdon SE Waldie B Woodward ND Wilman AH Tibbo PG. Procedural learning in first episode schizophrenia investigated with functional magnetic resonance imaging. Neuropsychology. (2011) 25:147–58. 10.1037/a0021222 21381822

[11] Reiss JP Campbell DW Leslie WD Paulus MP Ryner LN Polimeni JO. Deficit in schizophrenia to recruit the striatum in implicit learning: a functional magnetic resonance imaging investigation. Schizophr Res. (2006) 87:127–37. 10.1016/j.schres.2006.04.027 16814986

[12] Remillard G. The study of sequence learning in individuals with schizophrenia: a critical review of the literature. J Neuropsychol. (2013) 8:231–45. 10.1111/jnp.12022 23714117

[13] Berns GS Martin M Proper SM. Limbic hyperreactivity in bipolar II disorder. Am J Psychiatry. (2002) 159:304–6. 10.1176/appi.ajp.159.2.304 11823276

[14] McDowell I. Measuring Health : A Guide to Rating Scales and Questionnaires. New York, NY: Oxford University Press (2006).

[15] Young RC Biggs JT Ziegler VE Meyer DA. A rating scale for mania: reliability, validity and sensitivity. Br J Psychiatry. (1979) 133:429–435. 728692 10.1192/bjp.133.5.429

[16] Chrobak AA Hyla M Tereszko A Jeziorko S Siwek M Dudek D. Neuroprotective and neurotoxic effects of lithium: the role of different brain structures. Farmakoterapia Psychiatr Neurol. (2014) 2:113–20.15826742

[17] Buchanan RW Heinrichs DW. The Neurological Evaluation Scale (NES): a structured instrument for the assessment of neurological signs in schizophrenia. Psychiatry Res. (1989) 27:335–50. 10.1016/0165-1781(89)90148-0 2710870

[18] Gómez-Beldarrain M García-Moncó JC Rubio B Pascual-Leone A. Effect of focal cerebellar lesions on procedural learning in the serial reaction time task. Exp Brain Res. (1998) 120:25–30. 10.1007/s002210050374 9628400

[19] R Core Team. R: A Language and Environment for Statistical Computing. Vienna: R Foundation for Statistical Computing (2022).

[20] Leucht S Samara M Heres S Patel MX Furukawa T Cipriani A. Dose equivalents for second-generation antipsychotic drugs: the classical mean dose method. Schizophr Bull. (2015) 41:1397–402. 10.1093/schbul/sbv037 25841041 PMC4601707

[21] López-Larson MP DelBello MP Zimmerman ME Schwiers ML Strakowski SM. Regional prefrontal gray and white matter abnormalities in bipolar disorder. Biol Psychiatry. (2002) 52:93–100. 10.1016/s0006-3223(02)01350-1 12114000

[22] Zhao Q Li Z Huang J Yan C Dazzan P Pantelis C. Neurological soft signs are not “soft” in brain structure and functional networks: evidence from ALE meta-analysis. Schizophr Bull. (2014) 40:626–41. 10.1093/schbul/sbt063 23671197 PMC3984512

[23] Levinson AJ Young LT Fitzgerald PB Daskalakis ZJ. Cortical inhibitory dysfunction in bipolar disorder: a study using transcranial magnetic stimulation. J Clin Psychopharmacol. (2007) 27:493–7. 10.1097/jcp.0b013e31814ce524 17873683

[24] Nelson E Vinton D Berghorst L Towbin K Hommer R Dickstein D. Brain systems underlying response flexibility and bipolar adolescents: and event-related fMRI study. Bipolar Disord. (2007) 9:810–9. 10.1111/j.1399-5618.2007.00419.x 18076530

[25] Rowland LM Shadmehr R Krawitz D Holcomb HH. Sequential neural changes during motor learning in schizophrenia. Psychiatry Res. (2008) 163:1–12. 10.1016/j.psychresns.2007.10.006 18407471 PMC2562703

[26] Chrobak AA Bohaterewicz B Sobczak AM Marszał-Wiśniewska M Tereszko A Krupa A. Time-frequency characterization of resting brain in bipolar disorder during euthymia—a preliminary study. Brain Sci. (2021) 11:599. 10.3390/brainsci11050599 34067189 PMC8150994

[27] Caligiuri M Brown G Meloy M Eyler L Kindermann S. A functional magnetic resonance imaging study of cortical asymmetry in bipolar disorder. Bipolar Disord. (2004) 6:183–96. 10.1111/j.1399-5618.2004.00116.x 15117397

[28] Exner C Weniger G Schmidt-Samoa C Irle E. Reduced size of the pre-supplementary motor cortex and impaired motor sequence learning in first-episode schizophrenia. Schizophr Res. (2006) 84:386–96. 10.1016/j.schres.2006.03.013 16624528

[29] Chrobak AA Siuda K Tereszko A Siwek M Dudek D. Psychiatric disorders and the cerebellar structure and functions — an overview of the latest research. Psychiatria. (2014) 11:15–22.

[30] Chrobak AA Bohaterewicz B Tereszko A Krupa A Sobczak A Ceglarek A. Altered functional connectivity among frontal eye fields, thalamus and cerebellum in bipolar disorder. Psychiatr Pol. (2019) 133:1–11. 10.12740/pp/onlinefirst/104445 33038882

[31] Chen C-H Suckling J Lennox BR Ooi C Bullmore ET. A quantitative meta-analysis of fMRI studies in bipolar disorder. Bipolar Disord. (2011) 13:1–15. 10.1111/j.1399-5618.2011.00893.x 21320248

[32] Ong D Walterfang M Malhi GS Styner M Velakoulis D Pantelis C. Size and shape of the caudate nucleus in individuals with bipolar affective disorder. Aust N Z J Psychiatry. (2012) 46:340–51. 10.1177/0004867412440191 22368240 PMC3328643

[33] Chrobak AA Krupa A Dudek D Siwek M. How soft are neurological soft signs? Content overlap analysis of 71 symptoms among seven most commonly used neurological soft signs scales. J Psychiatr Res. (2021) 138:404–12. 10.1016/j.jpsychires.2021.04.020 33962127

[34] Chrobak AA Siwek GP Siuda-Krzywicka K Arciszewska A Starowicz-Filip A Siwek M. Neurological and cerebellar soft signs do not discriminate schizophrenia from bipolar disorder patients. Prog Neuropsychopharmacol Biol Psychiatry. (2016) 64:96–101. 10.1016/j.pnpbp.2015.07.009 26241859

[35] Chrobak AA Soltys Z Dudek D Siwek M. Neurological and cerebellar soft signs in bipolar disorder: the role of staging, type and history of psychotic symptoms. Prog Neuropsychopharmacol Biol Psychiatry. (2023) 121:110673. 10.1016/j.pnpbp.2022.110673 36349610

[36] Bolbecker AR Mehta C Johannesen JK Edwards CR O'Donnell BF Shekhar A. Eyeblink conditioning anomalies in bipolar disorder suggest cerebellar dysfunction. Bipolar Disord. (2009) 11:19–32. 10.1111/j.1399-5618.2008.00642.x 19133963

[37] Brown SM Kieffaber PD Carroll CA Vohs JL Tracy JA Shekhar A. Eyeblink conditioning deficits indicate timing and cerebellar abnormalities in schizophrenia. Brain Cogn. (2005) 58:94–108. 10.1016/j.bandc.2004.09.011 15878730

[38] Chrobak AA Rybakowski JK Abramowicz M Perdziak M Gryncewicz W Tereszko A. Vergence eye movements in bipolar disorder. Psychiatr Pol. (2020) 54:467–85. 10.12740/PP/ONLINEFIRST/105229 33038881

[39] Chrobak AA Rybakowski JK Abramowicz M Perdziak M Gryncewicz W Dziuda S. Vergence eye movements impairments in schizophrenia and bipolar disorder. J Psychiatr Res. (2022) 156:379–89. 10.1016/j.jpsychires.2022.10.042 36323140

